# The Jail Cure

**Published:** 1873-04

**Authors:** 


### THE JAIL CURE.

It ought not to be an unused fact that persons who are sent to jail very uniformly come out at the end of a few months, sleek and fat; so much so indeed, that often their best friends scarcely recognize them.

If any man wants to be broken of liquor drinking or smoking cigars, he has only to have himself sent to the penitentiary. The celebrated individual in Wall street, a few years ago, who "made paper" with such an unstinted hand, that is, forged names to notes and got the money on them, was proven on the trial never to have been seen without a cigar in his mouth; never to have been seen without complaining of a headache and had a physician in daily attendance, who was more successful in getting pay than in curing the "paper" maker; he diagnosed and prognosed the state of the man's pocket better than the state of his constitution, for on being asked by the court why he collected three hundred dollars a quarter in advance for infinitestimal pellets, he very gravely replied, he saw what would be the end of it, and that he had better secure himself while he could. This same "paper man" was sent to the penitentiary for four years; he fed on prison fare, plain and at regular times ; never was allowed so much as a single puff ; attended most assiduously to his duties as book-keeper ; gained fifteen pounds of flesh in less than three months ; served out his time ; left the prison in robust health, went over to Jersey, and is said to be one of the most efficient Sunday-school superintendents in all the "region round about."

Penitentiary board will cure any curable case of dyspeptia ; for it involves two essential things which are most potent remedies—plain food at five hours interval, nothing between, and steady muscular exercise. The remedy will be as efficient out of a penitentiary as in it, but not one man in a million will try it of his own accord. Plain food, taken at regular intervals, accompanied with moderate physical exercise, will cure any case of dyspeptia which can be cured at all ; no medicine ever has, or ever can cure that malady ; nothing can cure it, but a rested stomach and nourishing food.

